# Predicted Structures of *Ceduovirus* Adhesion Devices Highlight Unique Architectures Reminiscent of Bacterial Secretion System VI

**DOI:** 10.3390/v17091261

**Published:** 2025-09-18

**Authors:** Adeline Goulet, Jennifer Mahony, Douwe van Sinderen, Christian Cambillau

**Affiliations:** 1 Laboratoire d’Ingénierie des Systèmes Macromoléculaires (LISM), Institut de Microbiologie, Bioénergies et Biotechnologie (IMM), Aix-Marseille Université—CNRS, UMR 7255, 13009 Marseille, France; adeline.goulet@univ-amu.fr; 2 School of Microbiology & APC Microbiome Ireland, University College Cork, T12 K8AF Cork, Ireland; j.mahony@ucc.ie (J.M.); d.vansinderen@ucc.ie (D.v.S.)

**Keywords:** bacteriophages, lactic acid bacteria, AlphaFold, host adhesion device, secretion system VI

## Abstract

Bacteriophages, or phages, are sophisticated nanomachines that efficiently infect bacteria. Their infection of lactic acid bacteria (LAB) used in fermentation can lead to significant industrial losses. Among phages that infect monoderm bacteria, those with siphovirion morphology characterized by a long, non-contractile tail are predominant. The initial stage of phage infection involves precise host recognition and binding. To achieve this, phages feature host adhesion devices (HADs) located at the distal end of their tails, which have evolved to recognize specific proteinaceous or saccharidic receptors on the host cell wall. *Ceduovirus* represents a group of unique lytic siphophages that specifically infect the LAB *Lactococcus lactis* by targeting proteinaceous receptors. Despite having compact genomes, most of their structural genes are poorly annotated and the architecture and function of their HADs remain unknown. Here we used AlphaFold3 to explore the *Ceduovirus* HADs and their interaction with the host. We show that *Ceduovirus* HADs exhibit unprecedented features among bacteriophages infecting Gram^+^, share structural similarities with bacterial secretion system VI, and combine both saccharide and protein-binding modules. Moreover, we could annotate the majority of *Ceduovirus* genes encoding structural proteins by leveraging their predicted structures, highlighting AlphaFold’s significant contribution to phage genome annotation.

## 1. Introduction

Bacteriophages (phages), viruses of bacteria, exhibit a remarkable structural diversity. Typically, they consist of a genome-containing capsid, a connector, and a tail ending in a host adhesion device [1,2,3,4,5]. Tailed phages are categorized into three main morphologies: myophages, with long contractile tails; podophages, with short tails; and siphophages, with long non-contractile and flexible tails. While the taxonomy of phages is now solely based on sequence data and the *Caudovirales* order, and its component *Myoviridae*, *Siphoviridae*, and *Podoviridae* families have been abolished since 2021, the connotations of these morphological descriptions have merit in the context of structural studies [6]. Siphophages infecting Gram+ bacteria, such as members of the phyla Bacillota or Actinomycetota, contain a structural hub attached to the last hexamer ring of the major tail protein (MTP) [7,8]. This hub comprises a hexameric ring of the distal tail protein (Dit), connected on one side to MTPs and on the other to a trimeric tail-associated lysin protein (Tal). Functional domains involved in cell wall binding or hydrolysis may be inserted in this Dit-Tal structural hub. Evolved Dits, containing carbohydrate-binding modules (CBMs), have been identified in nearly all lineages of phages infecting Gram+ bacteria [9,10,11,12,13]. Tal proteins exhibit an even wider range of adaptations, including extensions of cell wall-degrading enzymes [4], CBMs [13,14], and receptor-binding domains [15,16]. In some instances, Tals are reduced to a simple structural domain, as seen in the *Lactococcus lactis* phage p2, and more generally, *Skunavirus* members [17]. This structural diversity of phage adhesion devices is also observed in phages infecting Gram-negative bacteria. For instance, while the coliphage T5 features a genuine Dit-Tal structural hub [18,19], phage lambda only has a minimal Dit and lacks the Tal [20]. Instead, a hetero-hexamer is bound to the Dit hexamer and serves as the receptor-binding protein (RBP) in place of the Tal.

*Ceduovirus* phages infect the Gram-positive species *L. lactis* and *Lactococcus cremoris* [21,22,23]. Based on their sequences, they have been classified into two groups: the c2 group with three phages, c2, M5938, and D4412, and the bIL67 group comprising seven phages, including bIL67, D4410, and M6162 [24]. The genes coding for structural proteins of both c2 and bIL67 phages have been partially annotated, notably leading to the identification of the major capsid protein (MCP), head maturation protease (HMP), major tail protein (MTP), tape measure protein (TMP), and the large terminase (TerL). Millen and Romero previously used HHpred to analyze two sets of three ORFs, L14_c2_, L15_c2,_ and L16_c2_, for the phage c2, and ORF34_bIL67_, ORF35_bIL67,_ and ORF36_bIL67_, for the phage bIL67 [24]. They identified the presence of CBMs, among which two CBMs related to the BppA protein of the LAB phage Tuc2009, suggesting that these ORFs may be involved in the recognition of cell wall polysaccharide (CWPS) during the initial, reversible step of host cell binding. Then, c2 and bIL67 recognize and irreversibly bind to membrane protein receptors: Pip for c2 and YjaE for bIL67 [21,24]. Millen and Romero also showed that these two sets of three ORFs mentioned above are involved in Pip/YjaE binding [24]. However, the canonical Dit and Tal have not been identified by sequence analysis in ceduoviruses, suggesting that the structure of their adhesion devices may differ from those of the siphophages studied thus far.

In this study, we aimed to gain structural insights into the adhesion devices of these enigmatic phages. Building on our previous work demonstrating the effectiveness of AlphaFold2 in predicting the structure of adhesion devices from diverse phages—including those infecting lactic acid bacteria (LAB) and mycobacteria [4,12,13,14,25,26], as well as large bacterial molecular machines [27], we conducted a similar structural prediction study on the adhesion devices of the ceduoviruses c2 and bIL67. This analysis was further extended to include additional structural proteins of these phages. The resulting structural insights were then leveraged to investigate potential interactions between the phages and their host receptors. Overall, our findings reveal structural features in *Ceduovirus* siphophages’ tail that bear architectural resemblance to the type VI secretion system (T6SS) puncturing device.

## 2. Materials and Methods

We performed predictions with AlphaFold3 on the DeepMind/Google server at https://alphafoldserver.com/ (accessed on 4 September 2025) [28,29]. The predicted local distance difference test (pLDDT) values of predicted structures were stored in the PDB files as B-factors and color-coded on the predicted structures. The pLDDT color-coded structures and the PAE color-coded domains were obtained from the ChimeraX option Tools/structure prediction/alphafold error plot. The final predicted protein or domain structures were submitted to the Dali [30,31] or FoldSeek [32] servers to identify the closest structural homologues in the PDB. Visual representations of the structures were prepared with ChimeraX [33,34].

## 3. Results

### 3.1. Predicted Structures of the c2 and bIL67 Host Adhesion Devices

**Figure 1:**
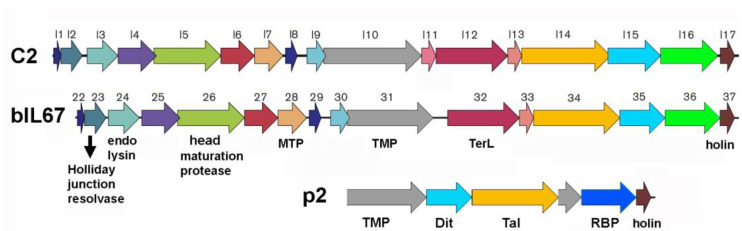
Schematic representation of the late genes of phages c2 and bIL67, together with the late genes of phage p2, between the TMP and the holin.

In lactococcal phages, such as the model phage p2, the genes encoding the components of adhesion devices are located between the genes encoding the *tmp* and the holin. The *dit* is typically the first gene downstream of the *tmp* and then commonly followed by the *tal* and *rbp* genes, plus, where relevant, accessory genes [4,8,17]. Therefore, using AlphaFold3 [28,29], we predicted the structure of the c2 and bIL67 unannotated proteins coded by genes between the *tmp* and the *holin,* which likely contain proteins of the host adhesion device, and searched for structural homologs using FoldSeek [32] and Dali [30,31] (Figure 1).

In both phages, the gene encoding the large terminase subunit (TerL; L12_c2_, ORF32_bIL67_), downstream of the *tmp*, is followed by the gene encoding the small terminase subunit (TerS; L13_c2_, ORF33_bIL67_) (Figure 1). The predicted structures of the three proteins encoded between this newly annotated *terS* gene and the *holin* display features commonly found in proteins that assemble phage adhesion devices or other phage-related macromolecular complexes, such as those of the T6SS.

#### 3.1.1. A Short CBM-Containing Tal/VgrG-like Protein

The L14_c2_ and ORF34_bIL67_ predicted structures each contain two domains. The N-terminal domains are well-defined, with pLDDT in the 80–100% range, and the PAE plot indicates low positioning errors (Figure S1A–F). Their structurally similar N-terminal domains are identified as CBMs (Figure S1, Table 1), which closely superimpose with each other (Figure S1G) and with the host-binding CBM of the BppA protein from the lactococcal P335 phage Tuc2009 adhesion device [35] (Table 1). This CBM assignment is consistent with previous observations by Millen and Romero [24]. The C-terminal domain of L14_c2_ is reasonably predicted, with pLDDT ranging from 70 to 90% and low global positioning errors (Figure S1B,C). In contrast, the C-terminal domain of ORF34_bIL67_ is poorly predicted, with pLDDT comprised between 50 and 70%, and PAE indicating at least three independently positioned modules (Figure S1E,F). The predicted C-terminal domain of L14_c2_ adopts the canonical fold of a Valine glycine repeat (VgrG), a component of the type VI secretion system (T6SS) [36], and of the Tal structural domain found in *L. lactis* phage p2 (Table 1). Therefore, the predicted structure of L14_c2_ supports an assignment as a VgrG/Tal-like protein of the c2 adhesion device. Comparable structural modularity of a structural domain and an adhesion extension was previously observed in the Tal of LAB phages that target polysaccharide receptors, such as those infecting *Streptococcus thermophilus* and *Oenococcus oeni*. The Tals of these phages contain CBMs within extended C-terminal regions, and/or lysin domains located either in their bulky N-terminal domain or within C-terminal extension [12,13]. Altogether, these results support the functional annotation of L14_c2_ and, to a lesser extent, ORF34_bIL67_ as the VgrG/Tal-like of the c2 and bIL67 phages.

#### 3.1.2. Hcp-like Proteins

The predicted structure of L15_c2_ exhibits three domains (Figure S2A): two poorly predicted N- and C-terminal domains (pLDDT between 50 and 70% and two independently moving domains) and a well-predicted middle domain (pLDDT 90–100% and low positioning errors (Figure S2B,C). In contrast, in L16_c2,_ the N- and C-terminal domains are well-predicted (pLDDT between 80 and 90%) and together form a unique independently moving domain, and the middle domain is also well-predicted (Figure S2D–F). The predicted structures ORF35_bIL67_ and ORF36_bIL67_ exhibit similar features compared to L15_c2_ and L16_c2_, respectively (Figure S2G–L). In L15_c2_ and ORF35_bIL67,_ the middle domain is identified by Foldseek as a BppA-like CBM domain, while FoldSeek returned similarities with an engo-glycosyl hydrolase, GH30, for the middle domains of L16_c2_ and ORF36_bIL67_ (Table 1).

Interestingly, the four N-terminal domains returned a Foldseek hit with the Hcp (Hemolysin-coregulated protein) of T6SS as structural homologs (Table 1). Hcps, the hallmark of T6SS, form hexamers that stack on top of each other to form the tube of T6SS, on which a VgrG trimer is plugged, the latter being involved in the puncturing of the target bacterium [36]. Therefore, we hypothesized that ORF35_bIL67_, ORF36_bIL67_, and L15_c2_, L16_c2_ may assemble Hcp-like hexameric rings, playing the role of hexameric Dit rings, to which a VgrG/Tal-like trimer could bind on one face and an MTP hexameric ring could bind to the other face.

#### 3.1.3. The ‘Core’ of the c2 and bIL67 Adhesion Device

To further test our hypotheses, we have predicted the structures of the ‘core’ of the c2 and bIL67 adhesion device, for which the VgrG/Tal-like (L14_c2_ and ORF34_bIL67_) and Hcp-like (L15_c2_, L16_c2_, ORF35_bIL67,_ and ORF36_bIL67_) proteins were devoid of their CBM, since these domains are usually peripheral [11,14], associated with one MTP (L7_c2_, ORF28_bIL67_) hexamer. We used the following protein combinations of homo- and hetero-hexamers of the Hcps: 6× MTP_c2_ + 3× L14_c2_ΔCBM + 6× L15_c2_ΔCBM (Figure S3A–C); 6× MTP_c2_ + 3× L14_c2_ΔCBM + 6× L16_c2_ΔCBM (Figure S3D–F); 6× MTP_c2_ + 3× L14_c2_ΔCBM + 3× L15_c2_ΔCBM + 3× L16_c2_ΔCBM (Figure S3G–I), and 6× MTP_bIL67_ + 3× ORF34_bIL67_ΔCBM + 6× ORF35_bIL67_ΔCBM (Figure S3J–L); 6× MTP_bIL67_ + 3× ORF34_bIL67_ΔCBM + 6× ORF36_bIL67_ΔCBM (Figure S3M–O); 6× MTP_bIL67_ + 3× ORF34_bIL67_ΔCBM + 3× ORF35_bIL67_ΔCBM + 3× ORF36_bIL67_ΔCBM (Figure S3P–R). We noticed that homo-hexamers form distorted assemblies with pLDDT in the 50–70 range and several independently moving domains (Figure S3A–F,J–O). In contrast, the hetero-hexamers exhibit lower pLDDT (80–90%) as well as unique globally moving Hcp/VgrG ensembles (Figure S3J–L,P–R).

**Figure 2:**
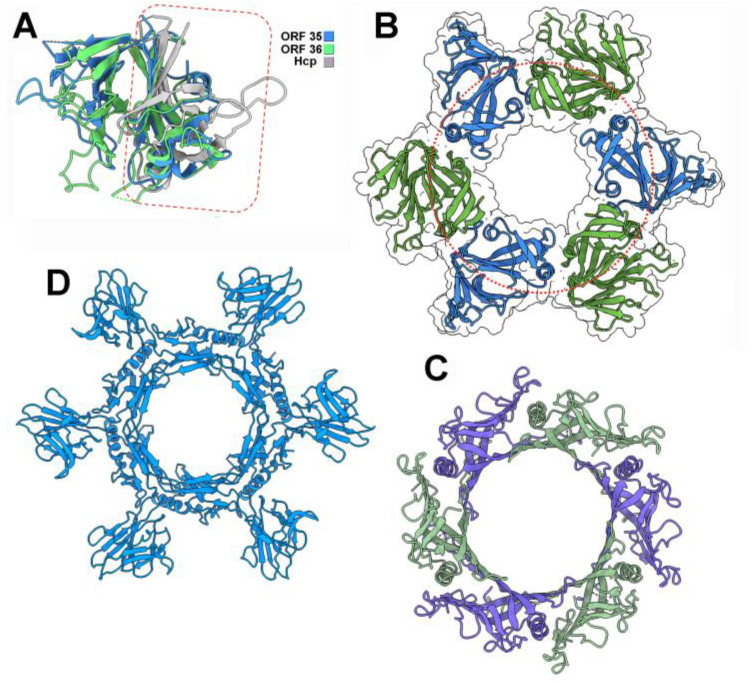
Structure analysis and comparison of the baseplate ORFs 35 and 36 components of phage bIL67. (**A**) Ribbon view of the superimposition of the N- and C-terminal domains of ORFs 35 and 36 and T6SS Hcp (PDB ID 7yw0). The Hcp-like N-terminal domains of the bIL67 ORFs are boxed in red. (**B**) Ribbon view of the N- and C-terminal domains of ORFs 35 and 36 forming the Dit-like ring. The Hcp-like N-terminal domains of the bIL67 ORFs are within the red circle. The C-terminal decoration is outside the circle. The CBM domains are not displayed. (**C**) Ribbon view of an Hcp hexamer from T6SS [38]. The Hcp monomers are identical but have been colored differently to facilitate the comparison with (**B**). (**D**) Ribbon view of a Dit hexamer from phage SPP1 [39]. Peripheral galectin-like domains are located outside the central ring.

The predicted structure of the MTP hexameric ring superimposes well on the experimental structures of typical phage MTP assemblies (Table 1). L15_c2_ and L16_c2,_ and ORF35_bIL67_ and ORF36_bIL67_, alternate within the rings and produce hexameric assemblies with a topology similar to that of Dit and Hcp rings, as indicated by the hits obtained using Foldseek-Multimer [37] (Table 1). In particular, their N-terminal domains, composed of two antiparallel β-sheets, form the inner part of the ring, which shares structural topology with T6SS Hcp and phage Dit hexamers (Figure 2, Table 1). For the C-terminal domains, in contrast, FoldSeek returns scattered non-specific hits.

**Figure 3:**
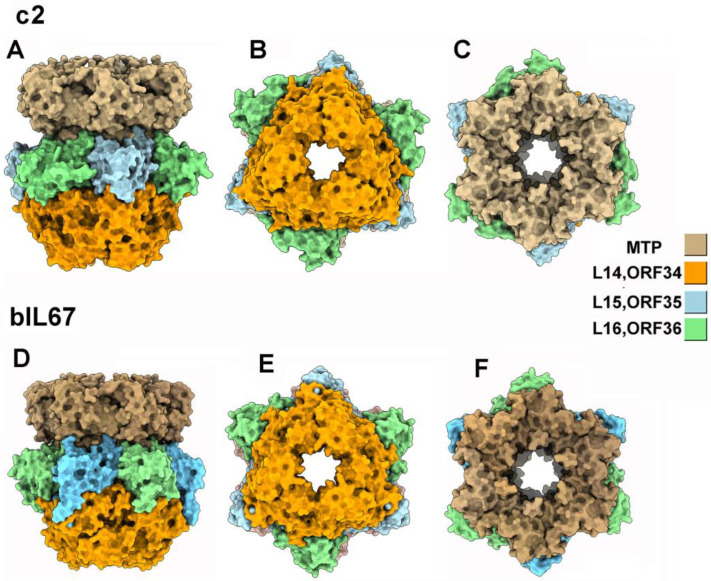
Structure of the baseplate core, CBM depleted, and MTP hexamer of phages c2 and bIL67. (**A**) Surface-side representation of the c2 core baseplate, including the Hcp-like hetero-hexamer, the VgrG-like trimer, and an MTP hexameric ring. (**B**) Same representation as in (**A**), but viewed from below. (**C**) Same representation as in (**B**), but viewed from the top. (**D**) Surface representation of the bIL67 core baseplate, including the Hcp-like hetero-hexamer, the VgrG-like trimer, and MTP viewed from the side. (**E**) Same representation as in (**D**), but viewed from below. (**F**) Same representation as in (**E**), but viewed from the top.

The trimers of L14_c2_ΔCBM and ORF34_bIL67_ΔCBM are bound to one side of these rings, while the MTP hexamers are bound to the other side (Table 1, Figure 3), with large interfaces between MTP and Hcp-like rings and between Hcp-like rings and Tal trimers (Table 2). In our predicted structures, the MTP and Hcp-like rings have an internal diameter of approximately ~40 Å, and the trimeric Tal partially obstructs the MTP-Hcp-like channel (Figure 3).

The trimers of ORF34_bIL67_ΔCBM and L14_c2_ΔCBM are structurally close to Tal trimers, such as that of the lactococcal phage p2 in its open conformation, as reported by Foldseek, and to T6SS VgrG trimers (Table 1). Altogether, these results show that (i) Hcp-like hetero-hexameric rings of ceduoviruses are topologically similar to the homo-hexameric rings of phage Dit proteins, such as that of the lactococcal phage p2 [17], and (ii) ceduovirus ORF34_bIL67_ and L14_c2_ trimers are topologically similar to T6SS VgrG trimers and phages’ short Tal trimers (Figure S5). Hence, the topology of the c2 and bIL67 ‘core’ adhesion devices resembles that of T6SS Hcp-VgrG complexes [36].

#### 3.1.4. The Complete c2 and bIL67 Adhesion Device

**Figure 4:**
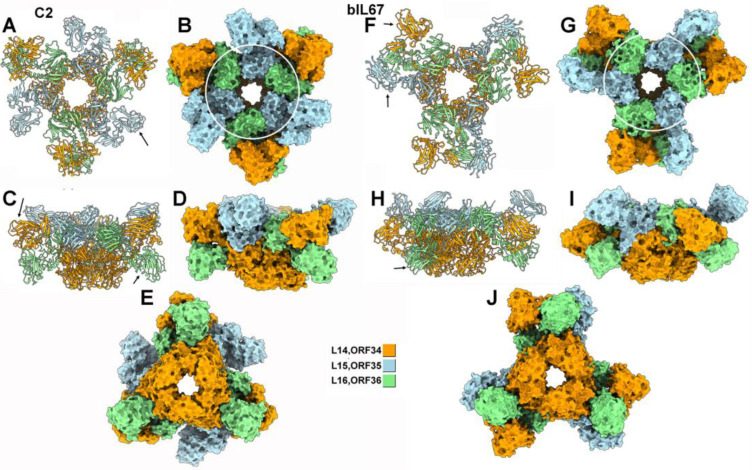
Structure of the baseplate of phages c2 and bIL67. (**A**) Ribbon representation of the c2 baseplate formed by L14–L16 viewed from top. (**B**) Same view as in (**A**), but with a surface representation. (**C**) Ribbon representation of the c2 baseplate viewed from the side. (**D**) Same view as in (**C**), but with surface representation. (**E**) Surface representation of the c2 baseplate viewed from below. (**F**) Ribbon representation of the bIL67 baseplate formed by ORFs34–36 viewed from the top. (**G**) Same view as in (**F**), but with surface representation. (**H**) Ribbon representation of the bIL67 baseplate viewed from the side. (**I**) Same view as in (**H**), but with surface representation. (**J**) Surface representation of the bIL67 baseplate viewed from below. Arrows show the CBMs positions. Circles in B and G delimitate the Hcp cores.

Lastly, we further explored the architecture of the c2 and bIL67 adhesion devices in the presence of the CBM by predicting the structures of multimers containing 3× L14_c2_ + 3× L15_c2_ + 3× L16_c2_, and 3× ORF34_bIL67_ + 3× ORF35_bIL67_ + 3× ORF36_bIL67_. It was not possible to add an MTP hexamer in the prediction, as the number of amino acids exceeded the AlphaFold limit of 5000. The pLDDT was better for the bIL67 baseplate, compared to c2 (Figure S5). Accordingly, the PAE of the bIL67 core identified a unique domain, in contrast with that of c2. As expected, the PAE identified each CBM as an independently moving unit (Figure S5). In both phages, all CBMs or GHs, which possibly have different saccharide specificities, are located at the periphery of the central ‘cores’ (Figure 4). These positions are compatible with a role in mediating the initial, reversible interactions with the host cell surface.

### 3.2. Mapping the Receptor Binding Site

Phages c2 and bIL67 interact with their host using the membrane-anchored protein receptors Pip and YjaE, respectively [40,41]. Pip and YjaE are homologs of YueB, the protein receptor of *Bacillus subtilis*-infecting phage SPP1 [42]. YueB is a major component of the type VII-like secretion system (T7SS-like) present in *Bacillota* [43], and this may also be the case for Pip and YjaE.

Millen and Romero postulated that the three ORFs assembling the c2 and bIL67 adhesion devices may be involved in Pip and YjaE binding and determining the lactococcal host range [24].

**Figure 5:**
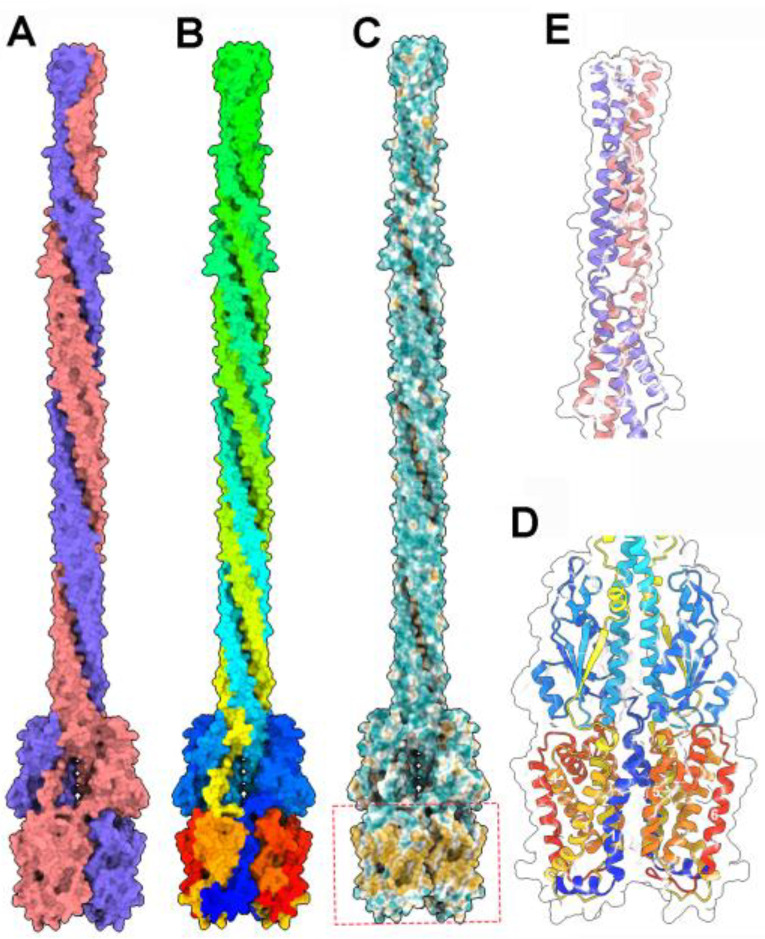
Structure prediction of the phage bIL67 receptor, the YjaE dimer. (**A**) Surface view of the YjaE dimer colored by monomer. (**B**) Same view, rainbow colored from the N-terminus (blue) to the C-terminus (red). Note that the N- and C-termini join together to form a compact domain. (**C**) Same view colored according to hydrophobicity. The N- and C-termini compact domain is boxed in red and colored brown, indicating a hydrophobic patch from trans-membrane helices. (**D**) Ribbon view of the rainbow colored YjaE N- and C-termini compact domain. Each monomer contains 6 trans-membrane helices, 1 from the N-terminus and 5 from the C-terminus. (**E**) Ribbon view of the tip of the YjaE dimer.

Therefore, with the aim of mapping the receptor-binding site on ceduovirus adhesion devices, we first predicted the structures of the full-length Pip and YjaE dimers to identify the region interacting with the phage. The structure of the YjaE dimer was predicted with high confidence, whereas the Pip dimer structure yielded low confidence scores and bizarre geometry. Consequently, we focused our analysis on the interaction between bIl67 and its receptor, YjaE. The YjaE dimer is a 360 Å-long rod in which each monomer folds upon itself, bringing the N-terminal and C-terminal regions into close proximity. The two monomers interact extensively along the entire length of the rod, resulting in a large interacting surface (Figure 5A,B). On one end of the rod, the YjaE N-terminal and C-terminal domains assemble altogether twelve hydrophobic α-helices likely inserting the receptor into the cell membrane (Figure 5C,D). On the opposite end of the rod, likely exposed at the cell surface, an α-helix bundle could be recognized by the bIL67 adhesion device (Figure 5E and Figure S6A).

**Figure 6:**
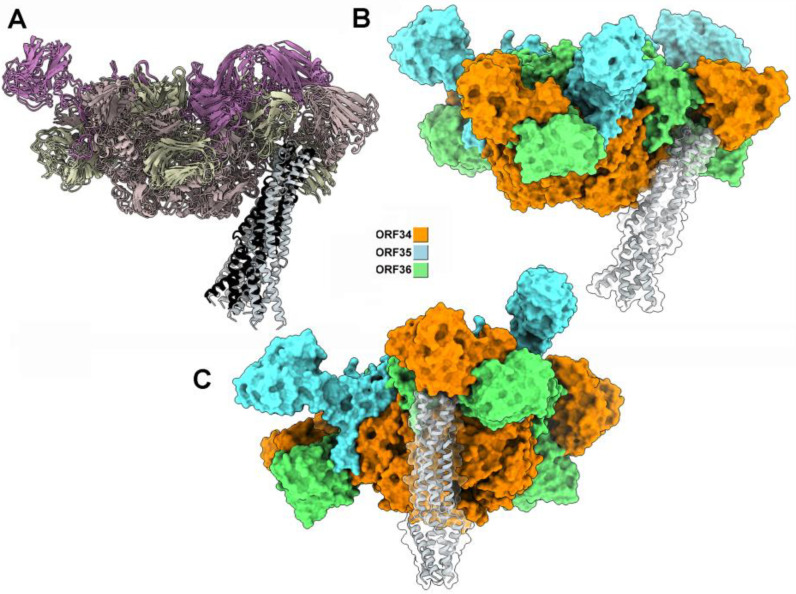
Putative structure of a complex between the baseplate of phage bIL67 with its receptor, YjaE. (**A**) Ribbon view of three converging solutions of the predicted complex. (**B**) Surface view of the first solution of the complex with the YjaE dimer tip (white). (**C**) Same view rotated by ~90°.

Then, we submitted the sequence of the YjaE tip (a dimer of residues 309 to 447) together with that of full-length ORF34_bIL67_, ORF35_bIL67,_ and ORF36_bIL67_, with a 3:3:3 stoichiometry for the structure prediction of the complex between the bIL67 adhesion device and its cell receptor (Figure S6B). Interestingly, three out of the five predicted structures revealed the same region of the bIL67 adhesion device interacting with YjaE. This potential receptor-binding site is formed by one VgrG-like monomer and one Hcp-like monomer (ORF36_bIL67_) (Figure 6A) and accounts for a total buried surface area of ~1000 Å^2^ (Figure 6B,C; Table 2C). Since AlphaFold relies solely on the evolutionary signals present in multiple sequence alignments to predict protein structures, the observed complementarity between the surface of the YjaE tip and the surface of the bIL67 adhesion device supports the identification of this bIL67 region as a possible receptor-binding site. However, the varying orientations of YjaE relative to the bIL67 adhesion device in the predicted structures (Figure 6A) prevent a detailed description of the structural and molecular determinants of this protein–protein interaction.

### 3.3. Quasi-Complete Annotation of the c2 and bIL67 Structural Genes

**Figure 7:**
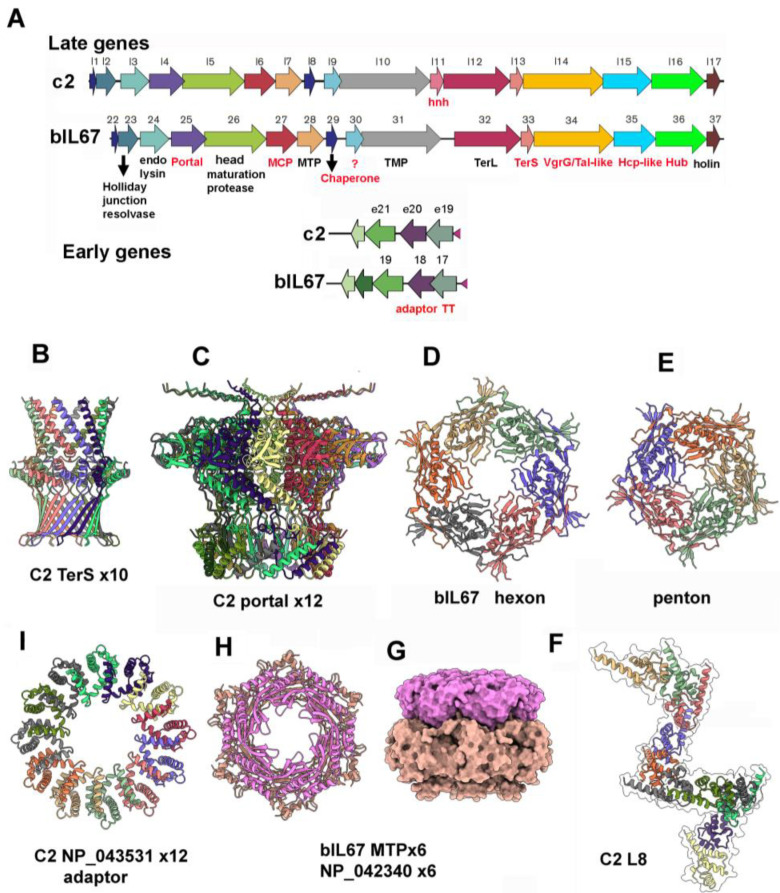
Assignment of the structural components besides the baseplate components using structure prediction. (**A**) View of the genomes: the previously assigned genes are in black, and the newly assigned in red. (**B**) Ribbon representation of a decamer of the C2 small terminase. (**C**) Ribbon representation of a dodecamer of the C2 portal. (**D**,**E**) Ribbon representation of a hexon and penton of the bIL67 MCP. (**F**) Ribbon representation of a multimer of the C2 TMP chaperone. (**G**) Surface view of docked hexamers of bIL67 MTP and early gene 17, the tail terminator. (**H**) Same as in (**G**), but ribbon representation. (**I**) Ribbon representation of a dodecamer of C2 early gene 18, the adaptor.

The annotation of the c2 and bIL67 structural genes in sequence databases is scarce. These include the large terminase (TerL; L12_c2_, ORF32_bIL67_), the major capsid protein (MCP; L152_c2_), the head maturation protease (HMP; ORF26_bIL67_), the tape measure protein (TMP; ORF31_bIL67_), the major tail protein (MTP; ORF28_bIL67_), the holin (L2_c2_/L17_c2_, ORF37_bIL67_), and the lysin (L3_c2_, ORF24_bIL67_) (Figure 7A,B). As for the genes annotated as minor structural proteins, L14_c2_, L15_c2_, L16_c2_, ORF34_bIL67_, ORF35_bIL67_, and ORF36_bIL67_, we could assign them a more precise function as the adhesion based on our AlphaFold3 predicted structures presented above.

Therefore, with the aim of providing a complete annotation of the *Ceduovirus* structural genes, we predicted the structures of all non-annotated genes encoding proteins of more than 80 amino acids and searched for structural homologs (Figure 7A). This led us to identify the c2 small terminase TerS coding gene *l13_c2_* downstream of the TerL coding gene *l12_c2_* (Figure 7A). L13_c2_ assembles as a decameric complex typical of these proteins (Figure 7B). We have identified the c2 portal protein coded by the *l4* gene, located between the genes encoding the MCP and endolysin. It assembles as the canonical dodecamer typical of phage portal proteins (Figure 7A,C). The bIL67 gene 27 codes for the MCP and forms the canonical penton and hexon (Figure 7D,E). Two ORFs between the MTP and the TMP are generally tail assembly chaperones in siphophages. The α-helical predicted structure of one of these ORFs in c2, L8_c2_, forms a spiral assembly reminiscent of the assembly formed by chaperones of the phages p2 and HK97 [44,45] (Figure 7F).

Interestingly, while the genes coding for the adaptor, stopper, and tail terminator are usually late genes located between the MCP and the MTP [4], we have identified the c2 *e19* and *e20* early genes and the bIL67 *orf17* and *orf18* genes encoding the tail terminator (NP_043530.1 for c2 and NP_042340.1 for bIL67) and adaptor (NP_043529.1 for c2 and NP_042342.1 for bIL67), respectively (Figure 7A). The bIL67 tail terminator forms the typical hexameric ring, but only when interacting with its MTP hexameric partner (Figure 7G,H), and the adaptor assembles as a canonical dodecamer (Figure 7I). Lastly, although we suspected that the stopper, which forms a hexameric ring interacting on one side with the adaptor and on the other side with the tail terminator, may be encoded by the c2 *e21* and bIL67 *orf19,* respectively, their predicted structure was not found to be similar to classical stoppers by FoldSeek or Dali, or by visual inspection. In phage JBD30, the neck lacks one of the typical three proteins between the portal and major tail protein, the tail terminator, or the stopper [46], and it was shown recently that phage P74–26 also lacks the stopper. This may also be the case for phages c2 and bIL67.

## 4. Discussion

The genome of ceduoviruses is compact, containing 37 to 39 ORFs, of which 16 to 17 are late genes that code for structural proteins expressed at the end of the phage replication cycle [24]. Compared to other lactococcal phages, their genome is highly rearranged. Notably, the *terL* and *terS* genes, typically found at the start of the late gene cluster, are located centrally. Even more unusual, two genes encoding phage neck components—*e19* and *e20* for c2, and *orf17* and *orf18* for bIL67—are not part of the late gene cluster but are instead situated at the end of the early gene set. This atypical organization underscores the unique genomic architecture of ceduoviruses. Furthermore, ceduoviruses are the only LAB-infecting phages that require interaction with a protein receptor for full attachment to their host [16]. Their adhesion devices exhibit distinctive features, including the absence of Dit and Tal proteins and of a bona fide receptor-binding protein. The Dit and Tal are replaced by a hetero-hexameric ring of T6SS Hcp-like proteins and a trimer of VgrG-like domains. The structural and functional parallels between T6SS and myophages, both acting as contractile devices, have been well-documented [47,48]. However, in siphophages, the tail is not contractile, and its structure resembles the T6SS central pilus [49]. Furthermore, the T6SS TssK trimer is structurally similar to receptor-binding proteins of skunaviruses [50].

**Figure 8:**
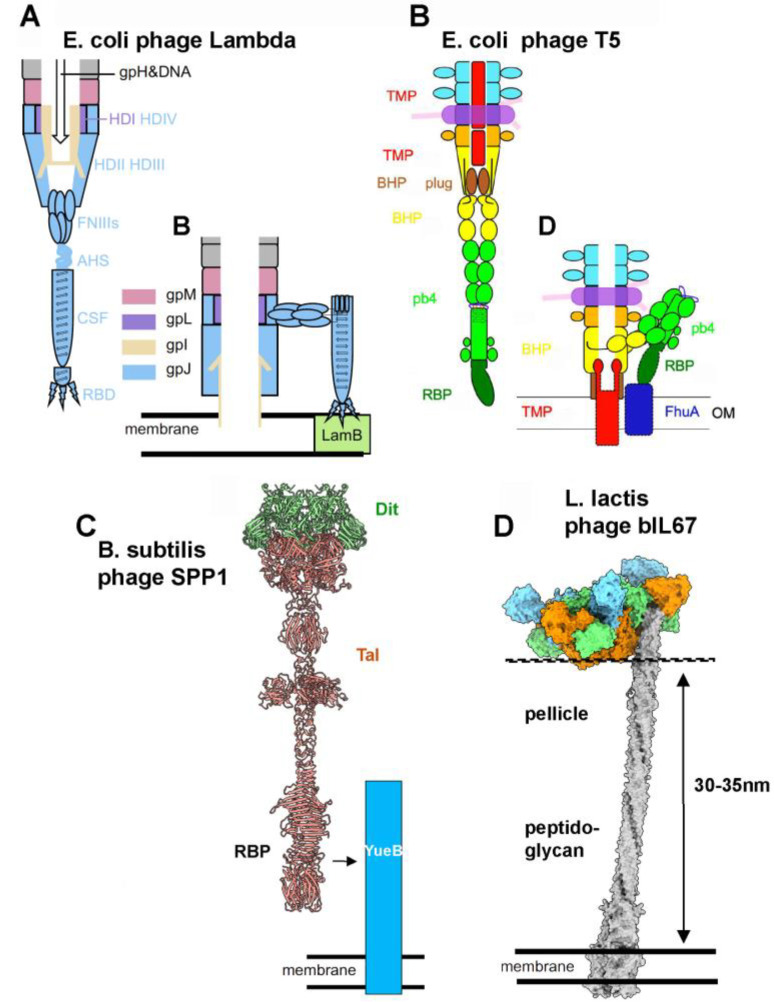
Mechanisms of phage/receptor attachments. (**A**) Schematic representation of phage lambda HAD before attachment to its membrane receptor LamB (**left**) and after (**right**). Adapted from [20]. (**B**) Schematic representation of phage T5 HAD before attachment to its membrane receptor FhuA (**left**) and after (**right**). Adapted from [19]. (**C**) Representation of the predicted structure of phage SPP1 HAD (Dit and Tal) and schematic interaction with its YueB receptor. (**D**) Predicted structure of the bIL67 HAD interaction with its YjaE receptor.

The attachment of siphophages to Gram^−^ and Gram^+^ protein receptors is well-documented at the structural level. The adhesion devices of coliphages such as λ and T5, which comprise a Tal and Tal-associated proteins, undergo conformational changes upon binding to their outer membrane receptors. These changes include (1) bending of the central tail fiber as it binds to the protein receptor and its lateral association with the receptor, (2) descent and opening of the Tal, and (3) subsequent binding of the TMP to the outer membrane (Figure 8A,B) [19,20,51,52]. For the Gram^+^-infecting phage SPP1, attachment to the *B. subtilis* cell surface involves recognition of the membrane-embedded receptor YueB, a component related to T7SS-like systems [16,43]. In this case, initial and reversible binding to cell wall teichoic acids facilitates subsequent recognition and irreversible attachment to YueB [53]. The SPP1 adhesion device is relatively simple compared to that of λ and T5 as it contains a non-evolved Dit and a long Tal [54]. Our structural prediction of the SPP1 Tal reveals the presence of a central CBM, which potentially interacts with cell wall teichoic acids, and a large C-terminal domain, which may serve as the YueB receptor. This Tal is also likely to undergo conformational changes upon receptor engagement, leading to its opening (Figure 8C).

In contrast to the rather elaborate adhesion devices described above, ceduoviruses possess a comparatively simpler adhesion device. Despite targeting a protein receptor, their adhesion devices feature nine CBMs. This suggests that the initial, reversible stage of infection involves a CBM-mediated attachment to saccharidic moieties on the host cell surface, similar to what has been shown for SPP1 [53]. A similar CBM-mediated host cell binding has recently been described for the *Mycobacterium smegmatis* phage Bxb1. While the CBMs of its Dit protein form a cage around the adhesion device in the free phage, they flatten against the adhesion device upon interaction with the mycobacterial cell wall [55]. Depending on the specificity of the CBMs for the encountered bacterial surface, two outcomes are possible: low specificity would result in phage dissociation before receptor engagement, whereas high specificity may provide sufficient dwell time for accessing and firmly binding to the receptor. Notably, the Gram^+^ bacteria cell wall can be quite thick, ranging from 30 to 100 nm, depending on the species [56]. In *L. lactis*, a cell wall thickness of 30–35 nm has been reported [57]. Given that the YjaE ectodomain is approximately 36 nm long, its tip is likely exposed at the cell surface, making it accessible for phage attachment (Figure 8D).

Structure predictions of the tip of YjaE ectodomain in complex with the bIL67 adhesion device converge to a unique binding site for the YjaE receptor, which supports the plausibility of this phage–host interaction. This hypothesis is further strengthened by spatial constraints imposed by the surrounding linkers and CBMs on the receptor-binding surface, and the complementary electrostatic surfaces of the adhesion device and cell receptor. However, how the ceduoviruses’ adhesion device penetrates the dense cell wall matrix and underlying peptidoglycan layer to reach the plasma membrane remains an open question.

## Figures and Tables

**Table 1:** Similarities of the baseplate components of phages bIL67 and c2 with structures deposited in the PDB. CBM: Carbohydrate binding module; EG: endoglucanase; GH: glycosyl hydrolase; Hcp: Hemolysin-coregulated protein; VgrG: Valine glycine repeat. Residues aligned: residues that are structurally aligned between the phage protein and the target protein in the PDB. Phages residues: the total number of protein residues involved in the comparison. Target residues: the total number of target residues involved in the comparison. rmsd: root mean square deviation. Z-value: quality indicator of Dali server: values below 4.0 are considered very weak.

**(A)**								
**bIL67**	**Target**		**Z-Value**	**rmsd Å**	**Residues** **Aligned**	**bIL67** **Residues**	**Target** **Residues**	**%ID**
34 CBM	5e7t	BppA CBM	11.7	3.3	173	234	286	16
35 CBM	5x7p	GH31	11.8	2.5	122	235	1247	13
36 CBM	2zxq	endo GH	12.4	2.5	133	227	1178	14
34 core	8gra	VgrG	5.8	5.0	202	375	613	7
35 core	7yw0	Hcp	3.8	3.2	86	179	120	9
36 core	7yw0	Hcp	5.6	3.1	83	186	120	18
36 core	35 core	Hcp	14.6	2.7	155	187	-	17
MTP	6v8i	MTP 80α	8.6	3.5	130	205	150	13
**(B)**								
**c2**	**Target**			**rmsd Å**	**Residues** **Aligned**	**c2** **Residues**	**Target** **Residues**	**%ID**
L14 CBM	5e7t	BppA CBM	12.1	4.1	193	259	286	19
L15 CBM	5e7t	BppA CBM	13.7	2.8	184	215	286	34
L16 CBM	2zew	Endo GH	10.6	2.5	132	246	147	11
L14 core	8gra	VgrG	6.7	5.1	231	380	445	6
L15 core	7yw0	Hcp	5.0	3.2	88	165	120	11
L16 core	7yw0	Hcp	4.9	3.3	87	188	120	8
L16 core	L15 core	Hcp	13.8	1.9	135	188	-	18
MTP	6v8i	MTP 80α	8.7	3.2	128	205	150	13
**(C)**								
**bIL67/c2 Core**	**Target**			**rmsd Å**	**Residues** **Aligned**	**bIL67** **Residues**	**c2** **Residues**	**%ID**
ORF34	L14	-	30.7	2.9	340	375	380	44
ORF35	L15	-	26.6	1.0	161	179	165	76
ORF36	L16	-	22.1	2.4	175	186	188	49

**Table 2:** Buried surface area (BSA) from the baseplate cores and MTP of phages c2 (**A**) and bIL67 (**B**). (**C**) Buried surface area between the baseplate (full-length ORFs) of phage bIL67 and the YjaE receptor tip. Values are in Å^2^.

(A)					(B)					(C)	
c2 Core	L14	L15	L16	MTP	bIL67 Core	ORF34	ORF35	ORF36	MTP	Full-Length ORF	YjaE
L14	1587	1510	1299	0	ORF34	1461	2049	1644	0	ORF34	574
L15	-	0	1262	866	ORF35	-	0	1377	992	ORF35	0
L16	-	-	0	820	ORF36	-	-	0	835	ORF36	510
MTP	-	-	-	2034	MTP	-	-	-	2072	TOTAL	1084
										YjaE	3037

## Data Availability

Coordinates of predicted structures are accessible on Zenodo (https://doi.org/10.5281/zenodo.17072877, accessed on 4 September 2025). Any additional information is available from the corresponding author upon request.

## Supplementary Materials

The following supporting information can be downloaded at: https://www.mdpi.com/article/10.3390/v17091261/s1, Figure S1: Structural prediction of monomers of phages bIL67 ORF34 and c2 L14; Figure S2: Predicted structures of monomers of phages c2 L15 and L16 and bIL67 ORF35 and ORF36; Figure S3: Representation and analysis of c2 and bIL67 core baseplate and MTP; Figure S4: Structures of the baseplates of phages p2 and bIL67 and of the puncturing device of T6SS. Figure S5: Predicted structures and analysis of c2 and bIL67 HAD; Figure S6: Structure and analysis of the YjaE dimer and its putative complex with bIL67 HAD.

## Abbreviations

The following abbreviations are used in this manuscript:

HAD	Host adhesion device
Tal	Tail-associated lysozyme
Dit	Distal tail
VgrG	Valine glycine repeat
Hcp	Hemolysin-coregulated protein
T6SS	Type VI secretion system
T7SS	Type VII secretion system
pLDDT	Predicted local distance difference test
PAE	Predicted aligned error
