# Supplementary material for: Predicted Structures of Ceduovirus Adhesion Devices Highlight Unique Architectures Reminiscent of Bacterial Secretion System VI
Source: Viruses. 2025 Sep 18;17(9):1261. doi: 10.3390/v17091261 (PMC12474359; doi:10.3390/v17091261)
Supplement: Supplementary file 1 [file viruses-17-01261-s001.zip › Supplementary-Figures-new.pdf]

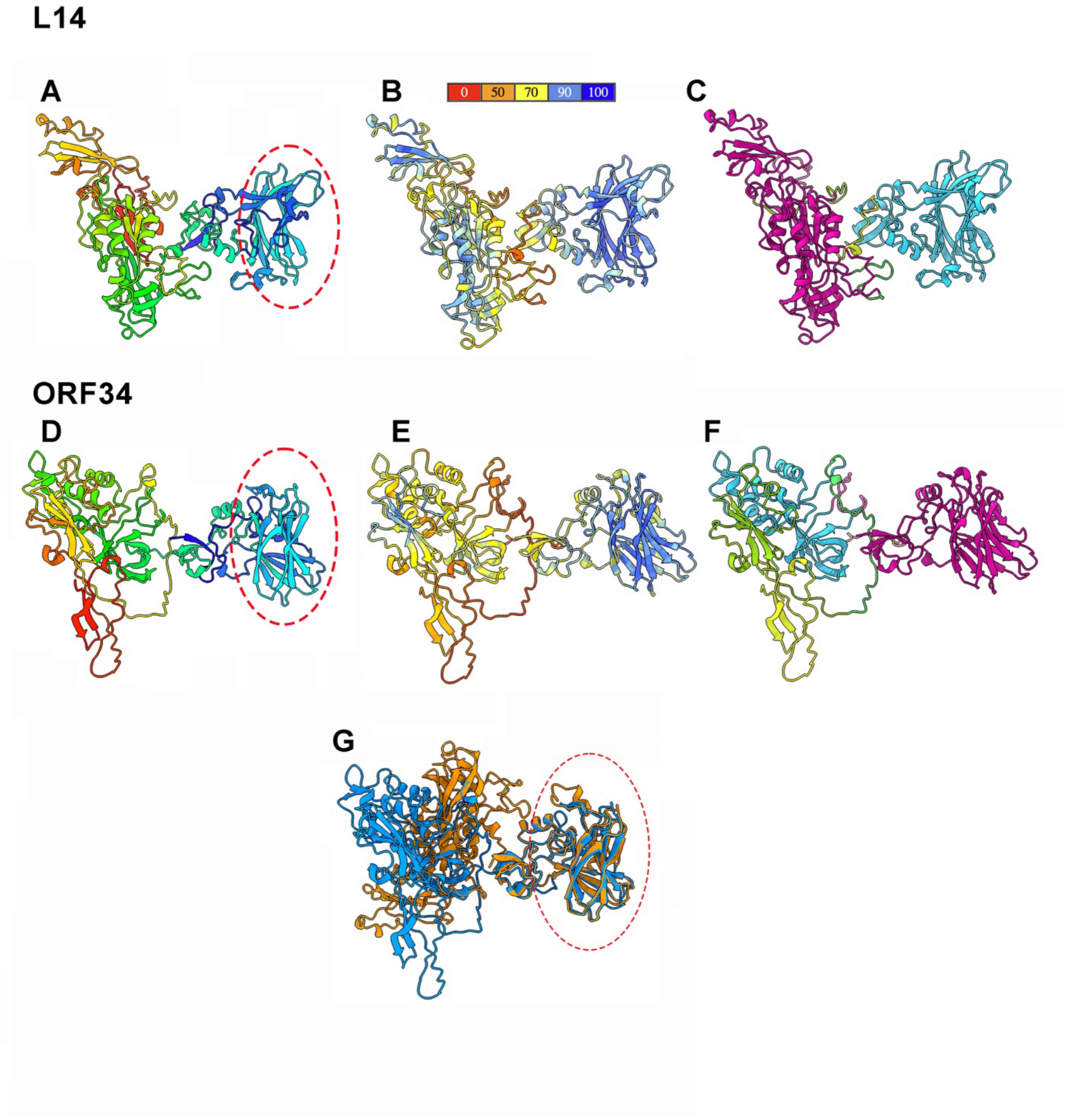

Figure S1: **Structural prediction of monomers of phages bIL67 ORF34 and c2 L14.** **a**/ Ribbon representation of c2 L14, rainbow colored (from Nter to Cter, blue to red). **b**/ same view but colored according to the pLDDT, from blue, good prediction, to red, poor prediction (see scale above). **c**/ same view but colored according to the PAE. Each color indicates an independently mobile domain. **d/** Ribbon representation of bIL67 ORF34, rainbow colored (from Nter to Cter, blue to red). **e**/ same view but colored according to the pLDDT, from blue, good prediction, to red, poor prediction). **f**/ same view but colored according to the PAE. **g**/ Superimposition of the N-terminal domains of L14 (orange) and ORF34 (blue) showing their perfect match. (**a**),(**d**): The N-terminal BppA-like CBM domains are circled red.

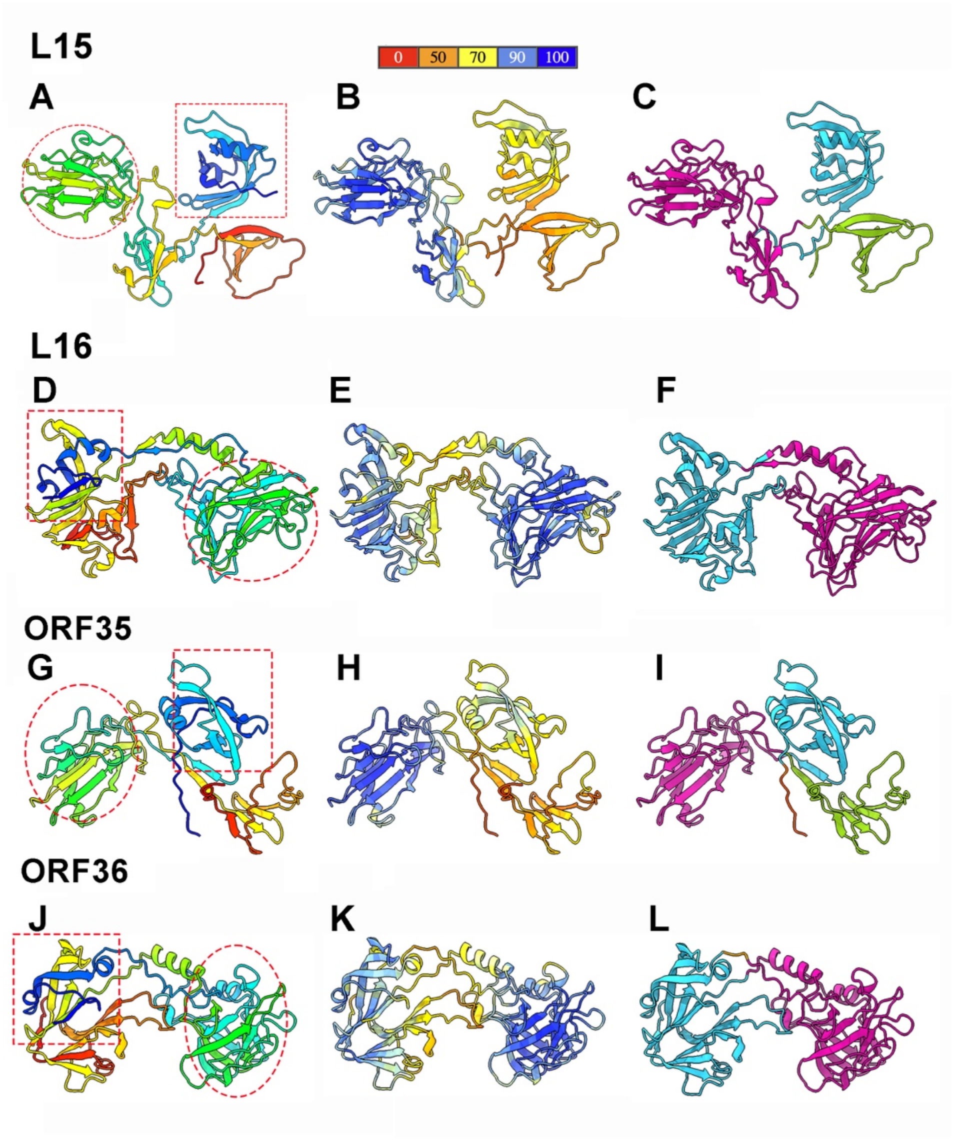

Figure S2: **Predicted structures of monomers of phages c2 L15 and L16 and bIL67 ORF35 and ORF36.** **a**/ Ribbon representation of c2 L15, rainbow colored (from Nter to Cter, blue to red). **b**/ same view but colored according to the pLDDT, from blue, good prediction, to red, poor prediction (see scale above). **c**/ same view but colored according to the PAE. Each color indicates an independently mobile domain. **d**/ Ribbon representation of c2 L16, rainbow colored. **e**/ same view but colored according to the pLDDT. **f**/ same view but colored according to the PAE. **g**/ Ribbon representation of bIL67 ORF35, rainbow colored (from Nter to Cter, blue to red). **h**/ same view but colored according to the pLDDT. **i**/ same view but colored according to the PAE. **j**/ Ribbon representation of bIL67 ORF36 rainbow colored (from Nter to Cter, blue to red). **k**/ same view but colored according to the pLDDT. **l**/ same view but colored according to the PAE. (a), (d), (g), (j): the N-terminal domains are squared red. The middle CBM domains are circled red.

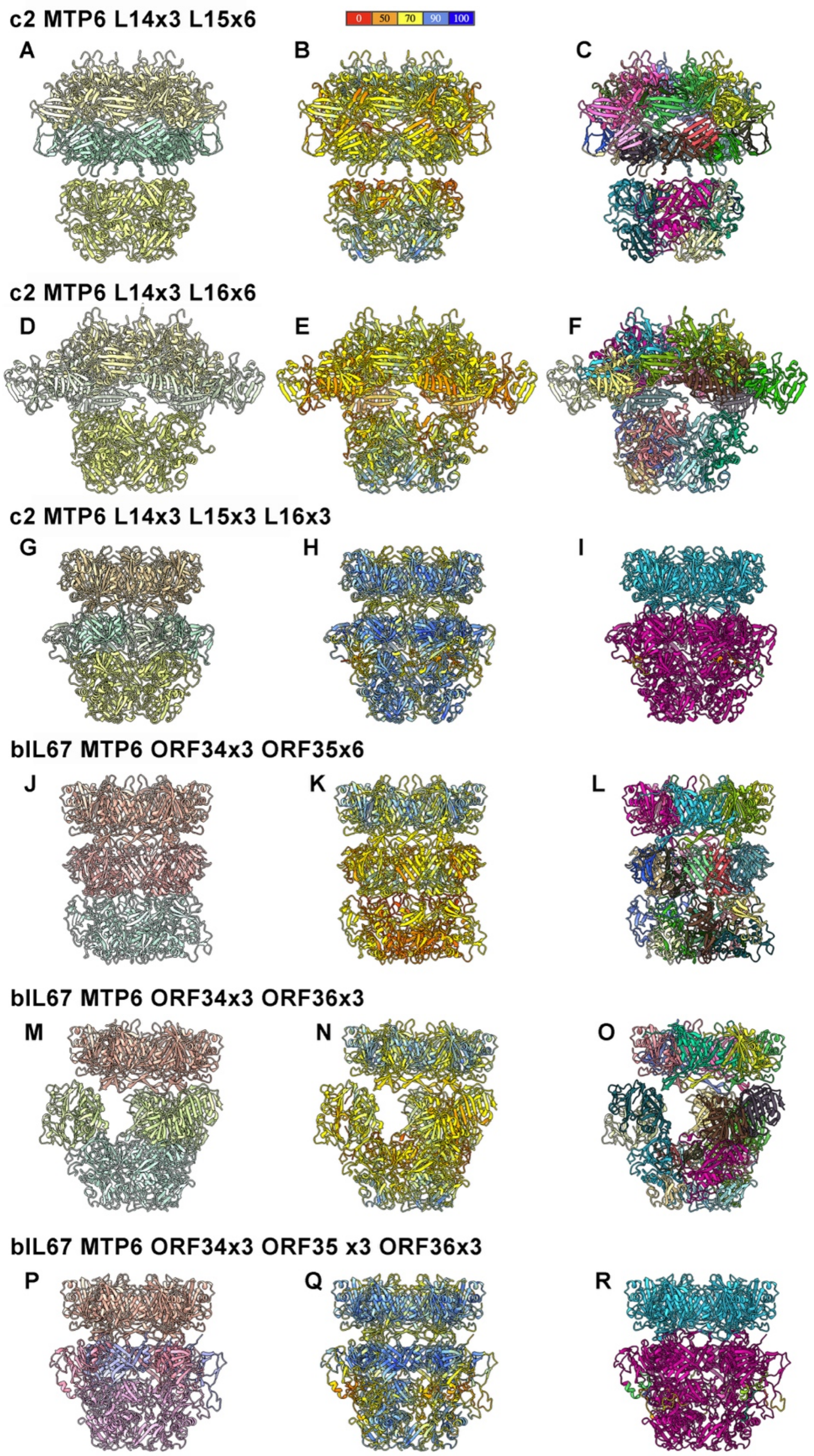

Figure S3: **Representation and analysis of c2 and bIL67 core baseplate and MTP**. The structures are viewed with the tail axis parallel to the plane. **(a), (d), (g), (j), (m), (p):** The structures (left) are colored according to their domains. **(b), (e), (h), (k), (n), (q)**: / same view but colored according to the pLDDT, from blue, good prediction, to red, poor prediction (see scale above). **(c), (f), (i), (l), (o), (r)**: same view (right) but colored according to the PAE. Each color indicates an independently mobile domain.

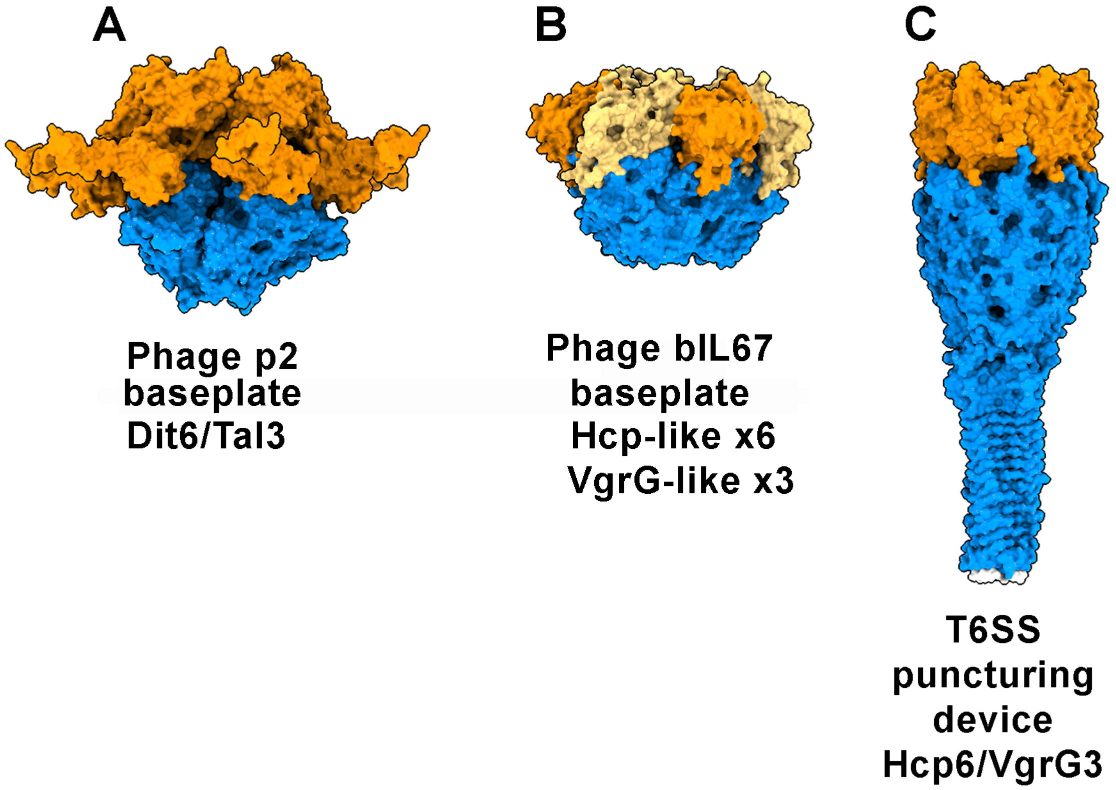

Figure S4: **Structures of the baseplates of phages p2 and bIL67 and of the puncturing device of T6SS**. **a/** Surface view of the baseplate from the lactococcal phage p2 {Sciara, 2010 #3475}. The Dit hexamer is orange and the Tal trimer is blue. **b/** Surface view of the baseplate from the lactococcal phage bIL67 (this work). The Hcp-like hetero hexameric complex is orange and yellow and the VgrG-like trimer is blue. **c/** Surface view of the puncturing device from a Bacillus fragilis T6SS {He, 2023 #3607}. The Hcp hexameric complex is orange and the VgrG trimer is blue. Note the long C-terminal extension compared to the structures in (a) and (b).

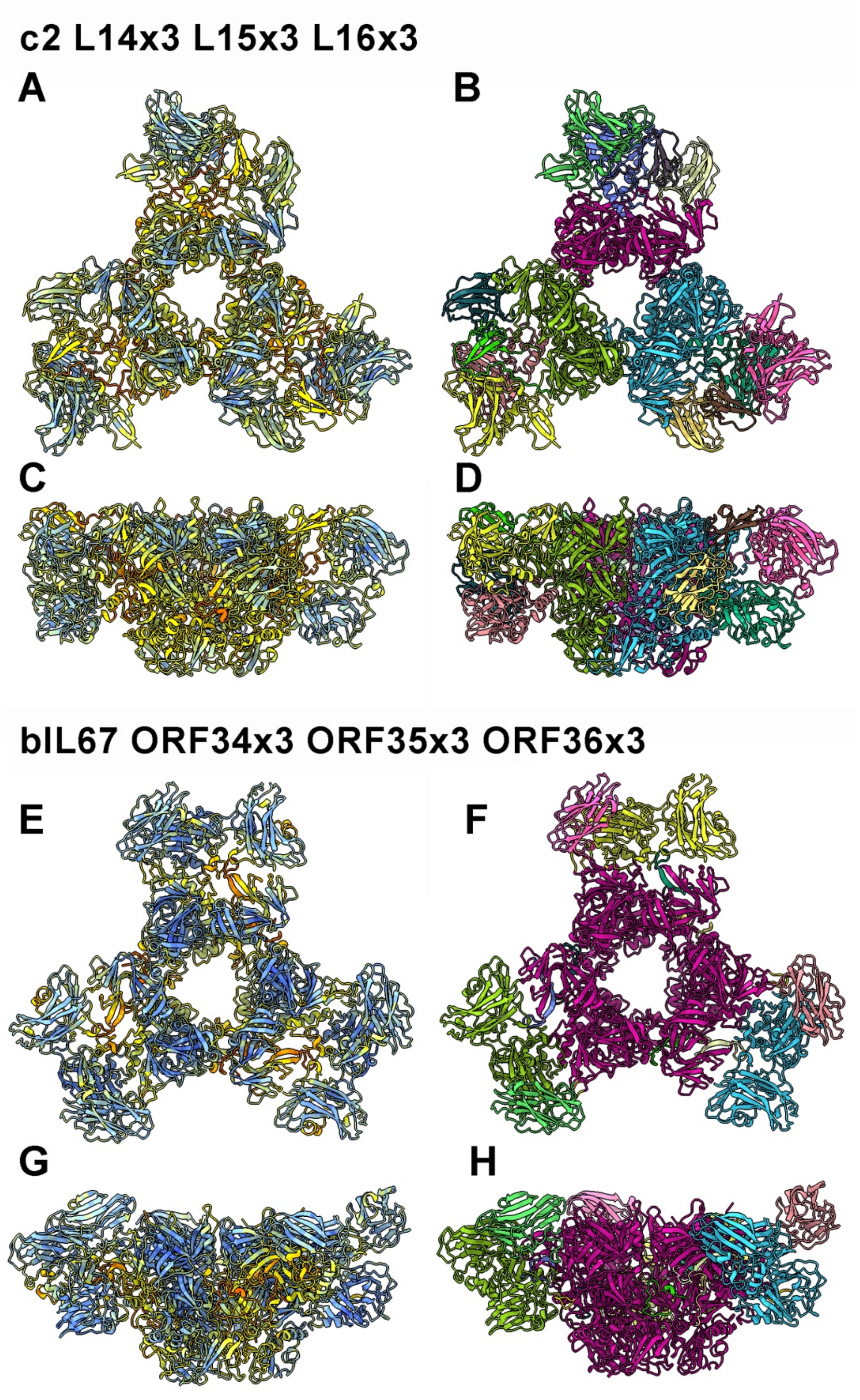

Figure S5: **Predicted structures and analysis of c2 and bIL67 HAD**. **a/, e/**: predicted hetero-hexameric complexes of Hcp-like and trimeric VgrG-like components colored according to the pLDDT, from blue, good prediction, to red, poor prediction. **c/**, **g/**: view rotated by 90° relative to (a), (e). **b/, f/**: same view as (a), (e) but colored according to the PAE. Each color indicates an independently mobile domain. d/, h/: view rotated by 90° relative to (b), (f).

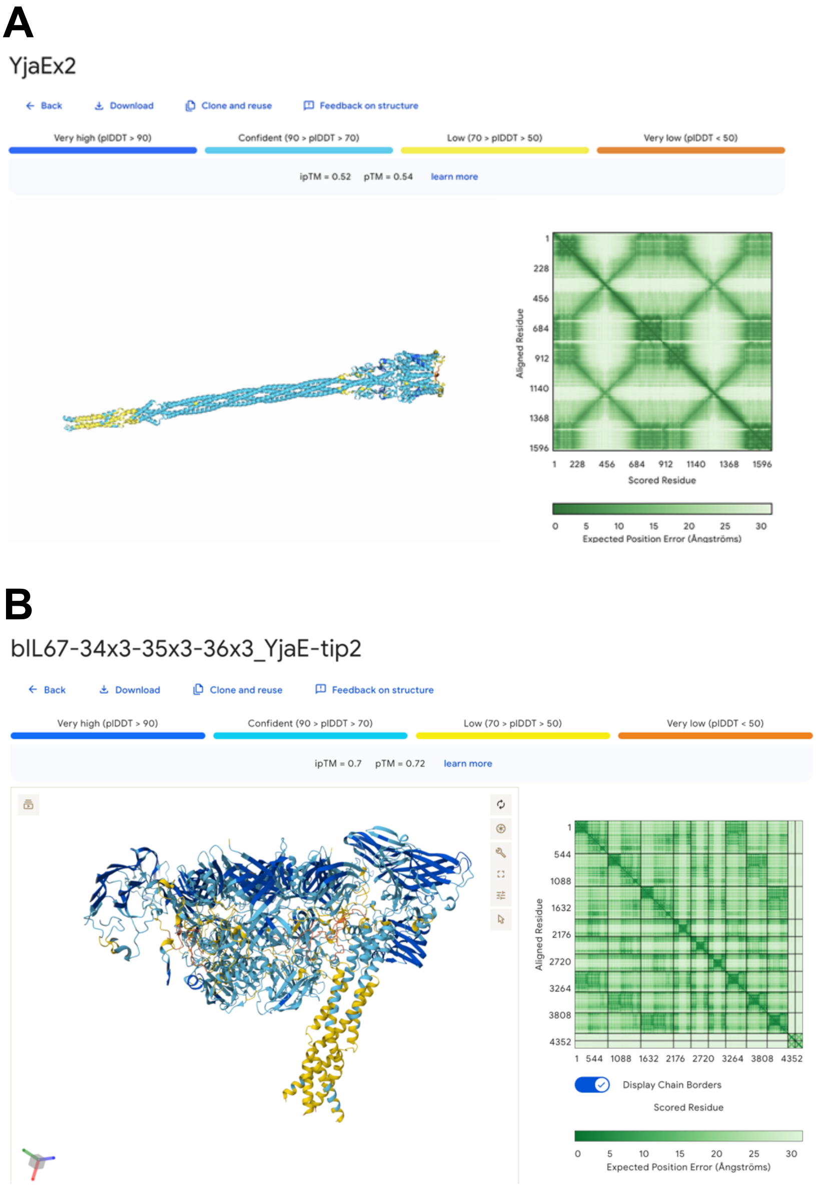

Figure S6: **Structure and analysis of the YjaE dimer (A) and its putative complex with bIL67 HAD (B)**. The structures (left) are colored according to pLDDT (good values are blue). The PAE are displayed on the right.
